# Salidroside regulates inflammatory pathway of alveolar macrophages by influencing the secretion of miRNA-146a exosomes by lung epithelial cells

**DOI:** 10.1038/s41598-020-77448-6

**Published:** 2020-11-27

**Authors:** Lanzhi Zheng, Jianming Su, Zhuoyi Zhang, Lu Jiang, Jinling Wei, Xiaoyang Xu, Shumin Lv

**Affiliations:** 1 Emergency Department, The First Affiliated Hospital of Zhejiang Chinese Medical University, Hangzhou City, 310006 Zhejiang Province China; 2 Department of Cardiology, The First Affiliated Hospital of Zhejiang Chinese Medical University, Hangzhou City, 310006 Zhejiang Province China

**Keywords:** Cell biology, Molecular biology

## Abstract

The purpose of this study was to explore the investigative mechanism of salidroside (SAL) on LPS-induced acute lung injury (ALI)/acute respiratory distress syndrome (ARDS). The exosomes from RLE-6TN are extracted and identified by transmission electron microscopy, particle size analysis and protein marker detection, and co-cultured with NR8383 cells. The ALI/ARDS model of SD rats was established by LPS (10 mg/kg) intratracheal instillation. Following a four-hour intratracheal instillation of LPS, 50 μl of RLE-6TN exosomes were injected through the tail vein. After that, SAL and miR-146a antagomir were injected into the tail vein for 72 h, respectively. As the changes of HE stain, body weight and ALI score are observed. The expression of miR-146a, TLR4, NF-kB, IRAK1, TRAF6 and their related proteins were detected by RT-PCR and Western blot, respectively. TNF-α, IL-6, IL-8 and IL-1 β inflammatory factors were detected by ELISA. The expression of miR-146a, NF-kB, IRAK, TRAF6 and related inflammatory factors in LPS-induced NR8383 was significantly higher than that in the control group, while SAL has greatly reduced the expression of TLR4 mediated NF-kB inflammatory pathway and related inflammatory factors. SAL can significantly improve the LPS-induced lung morphological abnormalities, slowed down the rate of weight loss in rats, and reducing the ALI score. The expression trend of NF-kB, IRAK, TRAF6 and related inflammatory factors in rats’ lung tissues was consistent with that in NR8383 cells. SAL has a protective effect on ALI/ARDS caused by sepsis, which is likely to be developed to a potential treatment for the disease. To sum up, this study provides a new theoretical basis for the treatment of ALI/ARDS with SAL.

## Introduction

Acute lung injury (ALI)/acute respiratory distress syndrome (ARDS) is an acute and progressive respiratory failure caused by severe infection, trauma, shock, inhalation of harmful gases and poisoning. While ALI and ARDS share similar pathophysiological changes, ALI in the severe stage can be defined as ARDS^1,2^. The main pathological feature of ALI/ARDS is pulmonary edema caused by increased pulmonary microvascular permeability, along with pulmonary interstitial fibrosis (PIF). The pathophysiological changes mentioned above mainly include reduced compliance of lung (CL), increased intrapulmonary shunt, disordered ventilation/blood flow ratio, and intractable hypoxemia. In the late stage, these are often complicated with organ function damage. Although researchers have studied ARDS intensively over the past 35 years, the pathogenesis of ARDS have not yet been defined completely^3^. Therefore, an in-depth study of the pathogenesis of ALI and search for new targets of gene therapy can provide some new insights in the clinical development of targeted drugs for ALI/ARDS treatment.

ALI/ARDS is associated with various clinical diseases, including both direct and indirect lung injury. There are more than 100 causes of ALI/ARDS, among which, inflammatory response plays an important role^4^. Due to the inflammatory mediators, the polymorphonuclear leukocyte (PMN) deformation ability decreases and becomes more likely to stay in the lungs under the action of chemokines before getting activated^5^. Activated PMN can lead to the release of a large number of inflammatory mediators, which can damage alveolar epithelial cells and ultimately lead to pulmonary edema and pulmonary ventilation dysfunction^6^. Alveolar macrophage (AM) is a resident phagocyte in the lung, and mainly distributed in the alveolar cavity and directly contacts with air^7^. miR-146a has been proved to be closely relevant to the occurrence and development of inflammations. Recent studies show that miR-146a can regulate the inflammatory response of THP-1 cells (human monocytes)^8,9^. It can also negatively regulate the expression of TLR4 pathway related molecules and downstream inflammatory factors. The expression of miR-146a is involved in the regulation of inflammatory response of AM, and its expression level is negatively correlated with the content of TNF-α mRNA.

Exosomes are nano particles (< 100 nm) secreted by cells, which can effectively deliver miRNA to receptor cells. They are gradually used as research methods to explore new therapeutic strategies and drug delivery vehicles. Therefore, it is of great significance to explore the role of exosomes in the delivery of miR-146a to AM and the regulation of inflammatory response.

At present, treating the primary disease, providing respiratory support and developing drug therapy are the main focuses of ALI/ARDS treatment. However, the lack of specific drugs and methods cannot achieve ideal therapeutic effects, thus leading to a high clinical fatality rate. Therefore, it is high time that the medical field should develop a treatment having fewer adverse reactions and more curative effects. In recent years, the development of traditional Chinese medicine’s treatment for lung injury and repair has been attracting increasingly more attention. Salidroside (SAL) is the main active component of Rhodiola, a perennial herb. Numerous experiments show that SAL can resist oxidation, inhibit the expression of inflammatory factors, and exert a significant protective effect on lung injury^10,11^. Guan et al. believe that SAL has a protective effect on ALI/ARDS induced by lipopolysaccharide (LPS) in animals^12^. Liu et al. have also found that SAL can reduce the expression of inflammatory factors HMGB1 and KRT-14 in blood, reduce pulmonary edema and increase oxygen partial pressure in ALI/ARDS rats, demonstrating an obvious therapeutic effect on ALI/ARDS^13^. Other evidences show that SAL not only directly inhibits the inflammatory activation of LPS-induced NR8383 cells, but also promotes the proliferation of RLE-6TN cells. It is involved in the regulation of alveolar epithelial cells on LPS-induced inflammatory activation of alveolar macrophages (AM)^14^. However, the specific cellular mechanism of anti-ALI/ARDS SAL is not clear. Therefore, this study aims to explore whether SAL can regulate the inflammatory pathway of AM by affecting the secretion of miRNA-146a exosomes in alveolar epithelial cells, in a bid to provide new clues for the treatment of ALI/ARDS.

## Methods

### Cell culture

The alveolar type II cell (RLE-6TN) was obtained from our laboratory and maintained in DMEM medium (Hyclone, USA) with 10% fetal bovine serum (FBS; Hyclone, USA), while the alveolar macrophages (NR8383) were obtained from our laboratory and cultured in RPMI-1640 medium (Hyclone, USA).

### Isolation and identification of exosomes containing miR-146a

The cell lines RLE-6TN were remained in 5% CO_2_ at 37 °C with DMEM containing 10% FBS without exosomes for 48 h. After that, cells and cell fragments were removed and supernatant was obtained by ultrahigh-speed centrifugation (3000×*g*, 10 min). Then the exosomes were extracted by SBI kit, which describes the specific extraction steps. The morphology of the exosomes was observed and photographed under an electron microscope, and the size of the exosomes was analyzed by particle size instrument. The expression of CD63 was detected by Western blot, and the protein was quantified by BCA method. The final concentration was 1 μg/L and the protein loading quantity was 10 μg, and the expression of miR-146a was detected by real-time PCR, and the concentration of RNA was 1 μg/L.

### Co-culture of exosomes and NR8383 cells

In order to check the uptake of exosomes in receptor alveolar macrophage NR8383, the exosomes from RLE-6TN were collected and labeled with PKH67 fluorescent cell labeling kit according to the manufacturer's instructions. Cells were cultured in 37 °C and 5% CO_2_ incubators for 24 h, and then 10 μl DMEM medium containing PKH67 labeled exosomes (100 μg/ml) or PBS solution was added. In addition, the green fluorescent miRNA was transfected into exosomes by the Exo-Fect Exosome Transfection Kit, and then the cells and exosomes were cultured in vitro for 2 h. After 4′,6-diamidino-2-phenylindole (DAPI) was used to dye the nucleus, before an anti-fluorescence quenching agent was added, and the image was taken by a confocal laser scanning microscope.

### Animal experiment

All animal protocols were approved by the Animal Experimental Ethics Committee of the First Affiliated Hospital of Zhejiang Chinese Medical University in accordance with the Guide for the Care and Use of Laboratory Animals issued by the Institute of Laboratory Animal Resources of the Life Science Committee of the National Research Council. 48 adult male SD rats weighing 160–200 g were fed in SPF animal room and allowed to drink freely. The experiment was carried out three days after that. All rats were randomly divided into 4 groups, 12 in each group. Rats were anesthetized by intraperitoneal injection of 10% chloral hydrate (0.003 ml/g), and LPS (10 mg/kg) was infused into the airway to establish the ALI model of SD rats with sepsis. The rat was placed in the head-up position. A white light in vitro irradiated its throat. Then its mouth was opened, with its tongue pulled out gently with forceps and fixed. The opening and closing of the rat's glottis was carefully observed in the light, while the corresponding LPS solution was drawn with a pipette. Targeting the opening and closing position of the glottis, the LPS solution was instilled. Then the bilateral nostrils of the rat was completely covered to block the upper part of its breath through the nostrils respiratory tract, so that ventilation could only pass through its mouth and throat to the trachea and the left and right main bronchi. After a four-hour intratracheal instillation of LPS, 50 μl exosomes were injected through the tail vein. The exosomes were isolated from the supernatant of 10^6^/ml RLE-6TN cell culture medium. After that, SAL (100 mg/kg by gavage) and miR-146a antagomir (10 nmol each rat 0.2 ml) were injected into tail vein for 72 h, respectively.

### Index evaluation of acute lung injury

Part of the left lung was fixed in 10% formaldehyde solution, and the histological changes were observed by paraffin embedding, sectioning and HE staining. The pathological score system of acute lung injury was established by American Thoracic Association (ATS). The scoring criteria are as follows: according to the severity of the lesions, the four indexes of alveolar congestion, hemorrhage, infiltration or aggregation of neutrophils in alveolar cavity or vascular wall, thickening of alveolar wall and (or) formation of hyaline membrane were rated in a 0-to-4 scale (0 refers to no or very slight lesions; 1 refers to mild lesions; 2 refers to moderate lesions; 3 refers to severe lesions; 4 refers to extremely severe lesions), with a total score of 16 points. The total score of four items represents the total score of lung injury.

### miRNA extraction and quantitative real-time PCR (qRT-PCR)

Total RNA was extracted using Trizol (Life Technologies), while cDNA was synthesized from 0.5 μg mRNA using a cDNA synthesis kit (Applied Biosystems, Japan). qRT-PCR was performed using an ABI Prism 7500 instrument (Applied Biosystems). Primers for miRNA-146a, TLR4, NF-kB, IRAK1, and TRAF6 were purchased from Sangon Biotech. The incubation was initiated at 37 °C for 15 min, followed by 95 °C for 2 min, 95 °C for 20 s, 56 °C for 20 s, 726 °C for 20 s and 40 cycles at 95 °C for 15 s, 55 °C for 15 min and 95 °C for 15 s. Each sample had three holes, and the experiment repeat 3 times. All primers are presented in Table S1.

### Western blot assay

Cells were collected and lysed in M-PER Mammalian Protein Extraction Reagent. All samples were normalized by their protein concentrations and separated in 10% SDS-PAGE gels and then transferred to membranes (Washington, NY) using the wet transfer blotting system (Hercules, CA). The antibodies used for Western blotting were: anti-CD63 (Abcam), anti-TRAF6 (Abcam), anti-TLR4 (Abcam), anti-NF-kB (Abcam), anti-IRKA1 (Abcam), and anti-GAPDH (Abcam), at a dilution of 1:1000. Then, protein bands were developed using ECL reagents, whose images were acquired using the ChemiDoc Imaging system. This western blot examination was repeated three times.

### Enzyme-linked immunosorbent assay (ELISA)

The serum levels of TNF-α, IL-6, IL-8 and IL-1β were determined by ELISA kits (Abcam, USA), following the manufacturer’s instructions.

### Statistical analysis

The SPSS 18.0 software was used for statistical analyses. All data were expressed as mean ± SD. Results were analyzed by one-way ANOVA with S-N-K multiple comparison test. *p* < 0.05 was considered to reflect a significant difference.

### Statement

All methods were carried out in accordance with relevant guidelines and regulations in the manuscript.

## Results

### Identification of exosomes and co-culture of exosomes and NR8383 cells

**Figure 1:**
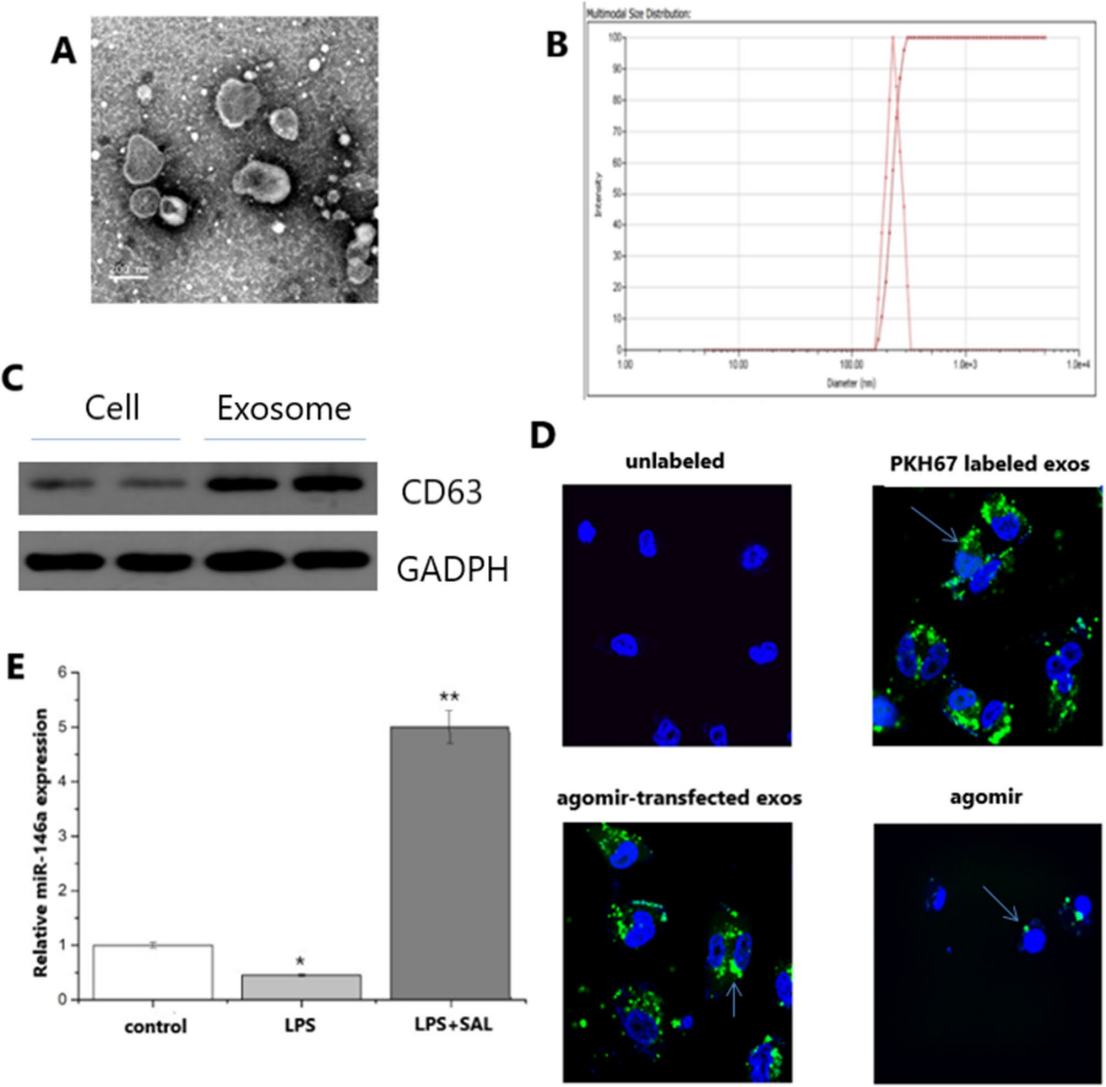
Identification of miRNA-146a exon and its transfer in NR8383 cells and the effect of SAL on the expression of miR-146a in LPS-induced RLE-6TN cells. (**A**) Electron micrograph of exosomes extracted by ultracentrifugation (bar = 200 nm). (**B**) Malvern size of exosomes. (**C**) Expression of CD63 in exosomes. (**D**) Laser confocal detection of exosomes transfer. (**E**) Effects of SAL and LPS on the expression of miR-146a in RLE-6TN cells.

The results of scanning electron microscopy and Malvern particle size of exosomes separated by high-speed centrifugation are shown in Fig. 1A,B. Most of these exosomes are 200–300 nm in diameter. Western blot shows that CD63 was enriched in exosomes, instead of in cells without exosomes (Fig. 1C). The exosomes isolated from RLE-6TN cells were labeled with PKH26 dye to check whether they could be absorbed by the receptor cell NR8383. In the confocal microscope, most of the receptor cells showed blue fluorescence and exosomes showed green fluorescence, proving that the exosomes transfected with fluorescent labeled miRNA could be transferred to the receptor cells (Fig. 1D).

### Effects of SAL on miR-146a expression in LPS-induced RLE-6TN cells

After pretreatment of RLE-6TN cells with SAL (50um) for 2 h, LPS (final concentration 0.1ug/ml) was used for further treatment, and exosomes were extracted after 24 h. The change of miR-146a expression was shown in Fig. 1E. Compared with the control group, the expression of miR-146a in LPS group was down-regulated (*P* < 0.05), while in LPS + SAL group was up-regulated (*P* < 0.01).

### Effects of SAL on miRNA-146a and TLR4 inflammatory pathway in LPS-induced NR8383 cells treated with RLE-6TN exosomes

**Figure 2:**
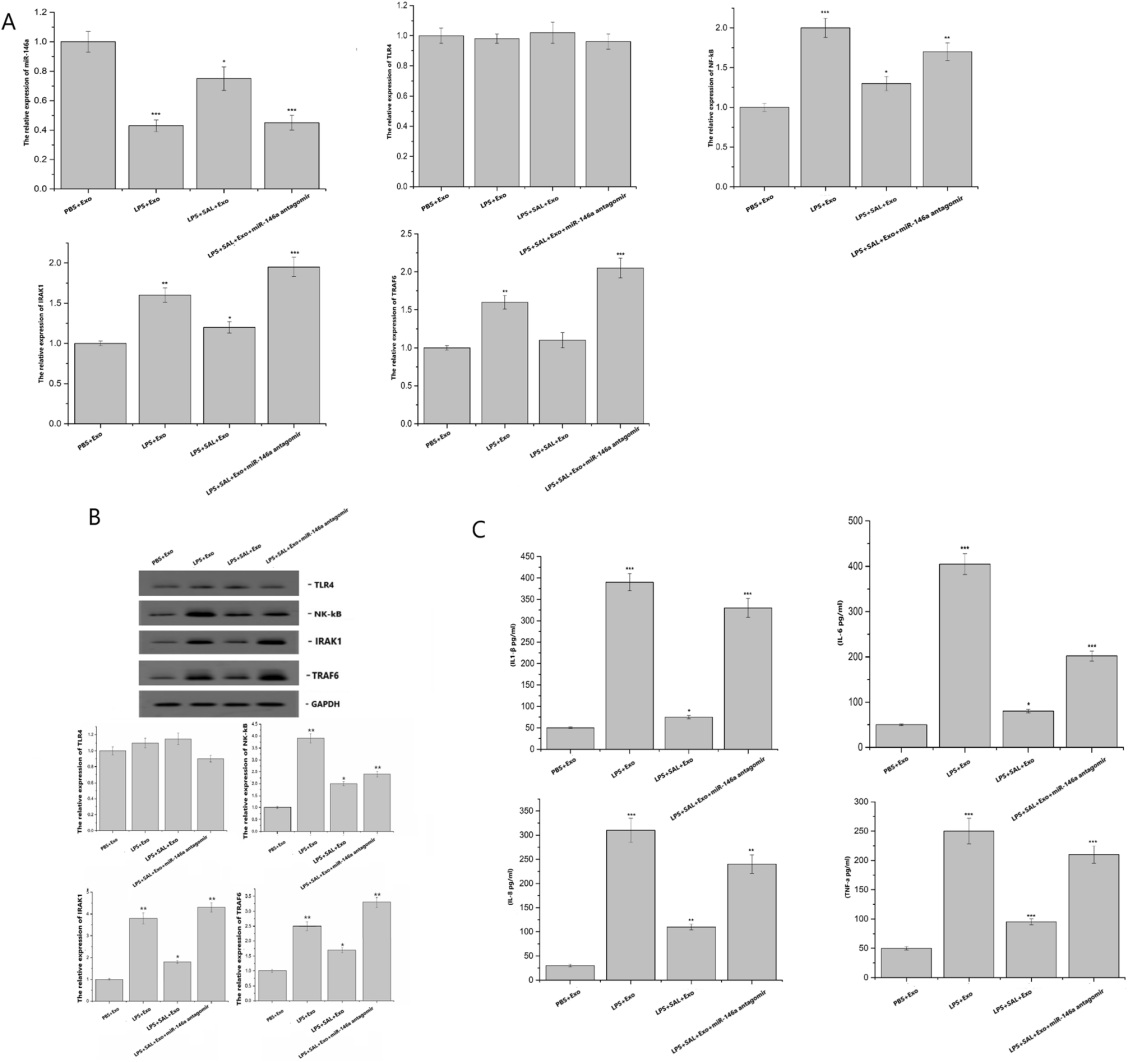
Effects of SAL on the expression of miR-146a and TLR4 related inflammatory factors in NR8383 cells. (**A**) Expression of miR-146a-TLR4 pathway was detected by RT-PCR. (**B**) Detection of target gene and signal pathway related gene expression by WB. (**C**) Detection of TNF-α, IL-6, IL-8 and IL-1 β inflammatory factors by ELISA.

Compared with PBS + Exo group, the expression of miRNA-146a was down-regulated in LPS + Exo group, LPS + SAL + Exo group and LPS + SAL + Exo + miRNA-146a antagomir group (*P* < 0.05). The expression of TLR4 showed no significant difference in LPS + Exo group, LPS + SAL + Exo group and LPS + SAL + Exo + miRNA-146a antagomir group, while the expressions of NF-kB and IRAK1 increased in LPS + Exo group, LPS + SAL + Exo group and LPS + SAL + Exo + miRNA-146a antagomir group (*P* < 0.05). The expression of TRAF6 showed no significant difference in LPS + SAL + Exo group, but significantly increased in PBS + Exo group and LPS + Exo group (*P* < 0.05) (Fig. 2A). The protein expressions of TLR4, NF-kB, IRAK1 and TRAF6 in each group were shown in Fig. 2B. The expression of TNF-α, IL-6, IL-8 and IL-1β inflammatory factors were shown in Fig. 2C. Compared with the PBS + Exo group, the contents of TNF-α, IL-6, IL-8 and IL-1β increased greatly in other groups (*P* < 0.05).

### Effects of SAL on LPS-induced acute lung injury in rats

**Figure 3:**
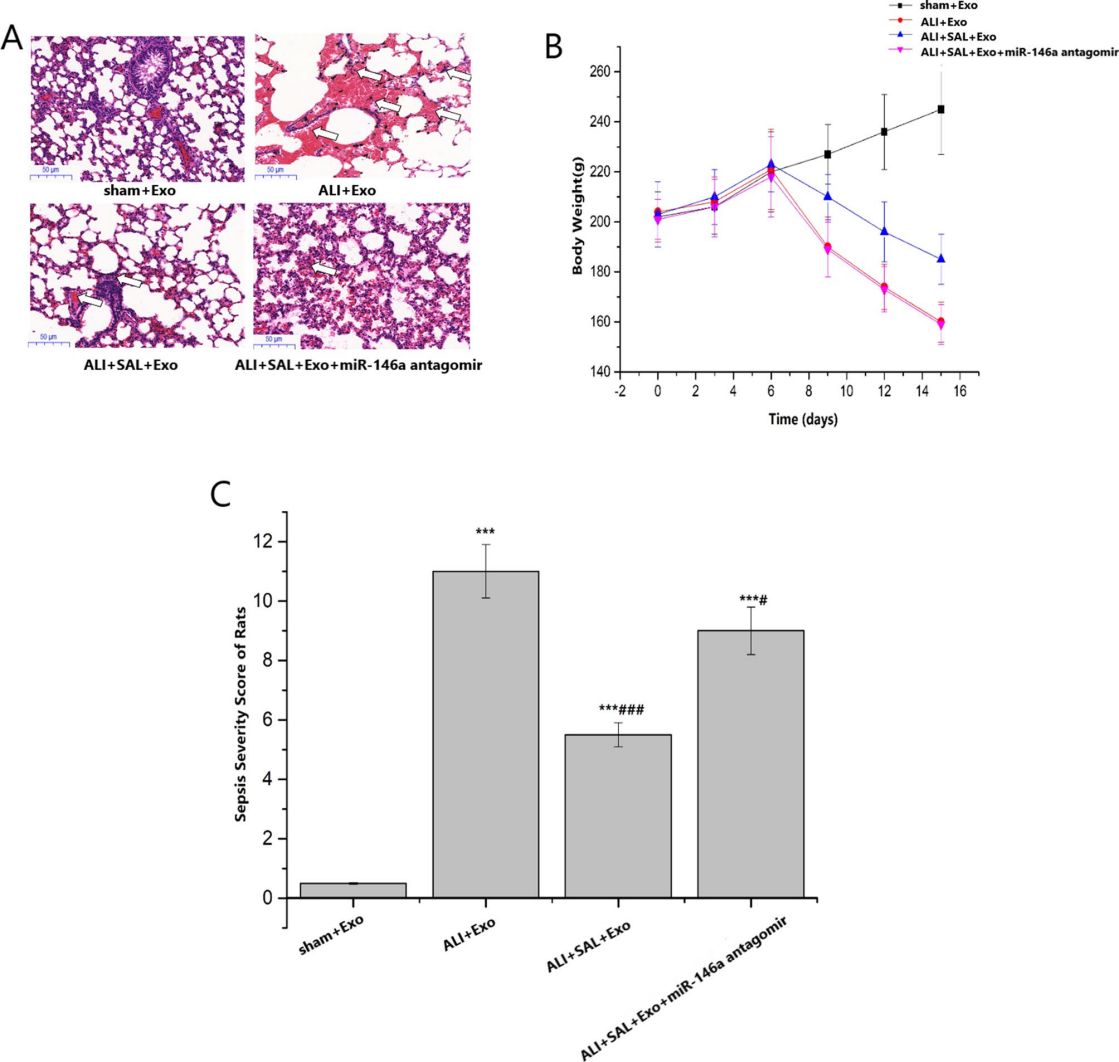
Evaluation indexes of acute lung injury in each group of rats. (**A**) HE staining results of lung tissues. (**B**) Trend of weight changes in rats. (**C**) ALI quantitative score of lung tissues.

As the results of HE staining of lung tissue showed, severe and extensive pulmonary edema, thickening of alveolar wall, local hemorrhage and neutrophil infiltration were observed in the rats of Sepsis lung injury group, compared with the sham + Exo group, while SAL could improve the morphological abnormality caused by LPS (Fig. 3A). In comparison with the sham + Exo group, the weight changes of rats in each group varied a lot. On the 15th day, the change trend of body weight was in such order: ALI + Exo group ≈ ALI + SAL + Exo + miR-146a antagomir group < ALI + SAL + Exo group < sham + Exo group, indicating that SAL had certain intervention effect on ALI (Fig. 3B). Compared with the sham + Exo group, the ALI scores of each group significantly increased. Compared with the ALI + Exo group, the scores of ALI + SAL + Exo group and ALI + SAL + Exo + miR-146a antagomir group were dropped significantly (*P* < 0.05) (Fig. 3C).

### Effects of SAL on miRNA-146a and TLR4 inflammatory pathway in lung tissues of rats with LPS-induced acute lung injury

**Figure 4:**
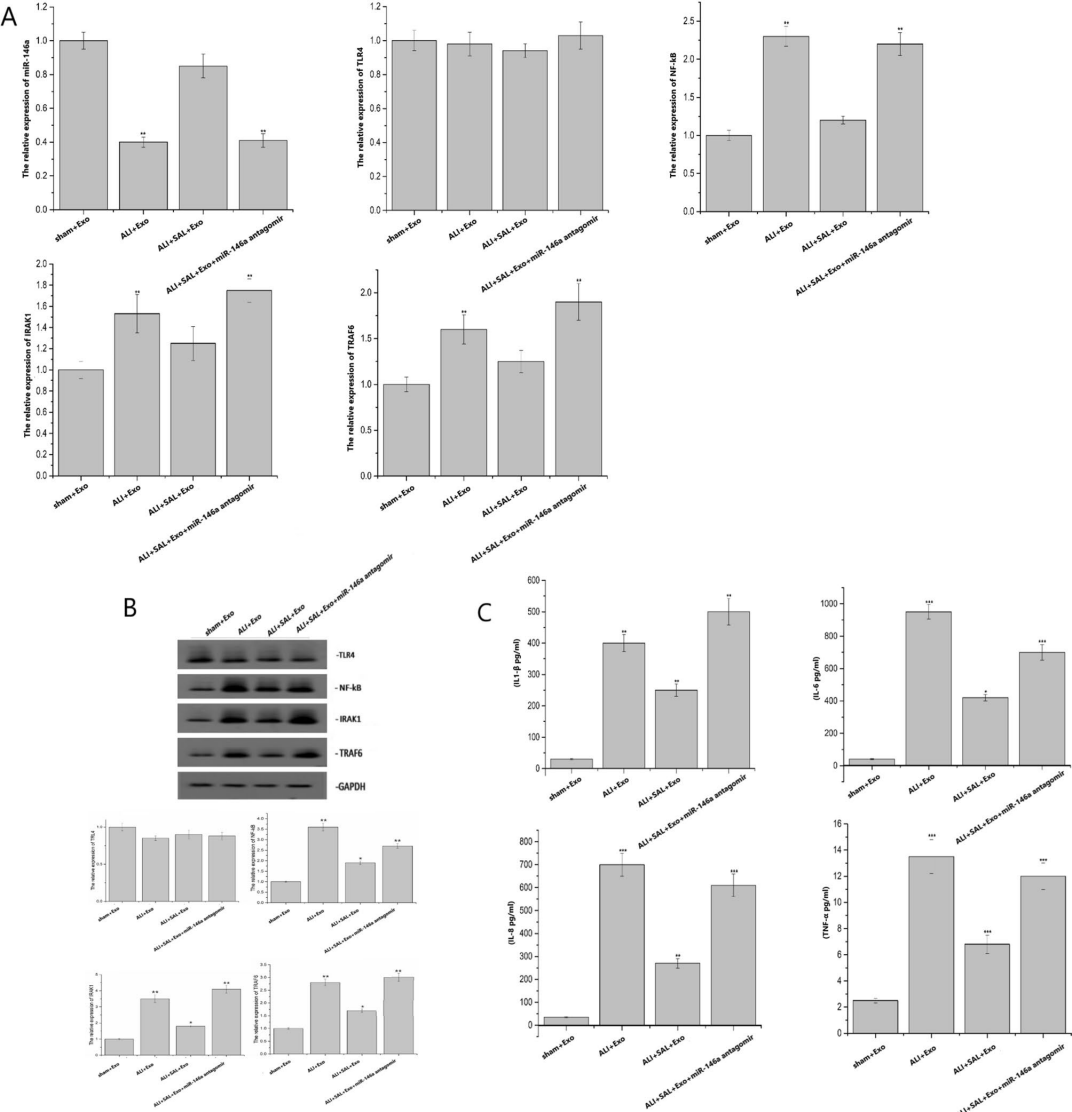
Effects of SAL on the expression of miR-146a and TLR4 related inflammatory factors in lung tissue of rats. (**A**) Expression of miR-146a-TLR4 pathway was detected by RT-PCR. (**B**) Detection of target gene and signal pathway related gene expression by WB. (**C**) Detection of TNF-α, IL-6, IL-8 and IL-1 β inflammatory factors by ELISA.

Compared with the sham + Exo group, the expression level of miR-146a in other each group decreased significantly (*P* < 0.05), and the expression of TLR4 showed no significant difference in other groups. The expression of NF-kB, IRAK1 and TRAF6 was significantly up-regulated in ALI + Exo group and ALI + SAL + Exo + miR-146a antagomir group (*P* < 0.05) (Fig. 4A). The expressions of TLR4, NF-kB, IRAK1 and TRAF6 proteins in the lung tissues of each group was shown in Fig. 4B, while Fig. 4C showed the expression of inflammatory cytokines in rats with lung injury in each group. Compared with the sham + Exo group, the expressions of IL-1, TNF-α, IL-6 and IL-8 in the ALI + Exo group, ALI + SAL + Exo group and ALI + SAL + miR-146a antagomir group were significantly up-regulated.

## Discussion

ALI/ARDS refers to acute and progressive respiratory failure caused by a variety of pathogenic other than cardiogenic factors. Accumulation and activation of central granulocytes in the lung, release of inflammatory mediators and oxidative stress are important pathogenesis of ALI/ARDS. Studies have shown that miRNAs are richly expressed and highly sensitive in the body, which can participate in the innate immune response of the body and regulate the inflammatory response as immune regulatory factors. By targeting miRNA related to inflammation it may therefore serve as a new and effective method to treat ALI/ARDS caused by sepsis.

miR-146a is one of the negative post-transcriptional regulators of immune cells transduced by TLRs (TLR2, TLR4)^15,16^. The results of Yamasaki et al. show that miR-146a can complement and bind with the 3′- UTR of TRAF6, IRAK1 and NF-kB mRNA, inhibit their expression, and then weaken the downstream signal transduction^17^. Therefore, miR-146a may be a negative regulator of TLR receptor and related cell signaling pathways, which is consistent with the results of this study.

The exosome are microbubbles composed of bilayer lipid membrane, playing a crucial role in the development of pathophysiology by transmitting specific information (such as miRNA, DNA, protein, etc.) to receptor cells, and mediating cell communication and information transmission^18–21^. In this study, the exosomes secreted by alveolar epithelial cells are successfully extracted by differential ultracentrifugation. The results of TEM, Western blot and particle diameter are consistent with the characteristics of exosomes. For a long time, it has been confirmed that exosomes mediate the connection between alveolar epithelial cells and immune cells under stress. Lee et al. have found that acid-induced alveolar epithelial microbubbles can mediate the migration of macrophages, and under hyperoxia condition, the cell microbubbles derived from pulmonary epithelial cells can activate macrophages to mediate inflammatory response through microRNA^22,23^. Furthermore, Moon et al. have found that exosomes derived from alveolar epithelial cells induced by hyperoxia can activate macrophages by transferring Caspase-3, which can lead to pulmonary inflammatory response^24^. Nevertheless, in sepsis lung injury, the effect of exosomes produced by alveolar epithelium on AM has not been reported. However, in this study, it was found that the secretion of miR-146a exosomes by lung epithelial cells could regulate the inflammatory pathway of alveolar macrophages.

Rhodiola is an important Tibetan medicine. Previous studies have shown that rhodiola rosea has anti-oxidation, anti-fatigue and immune regulation effects, which may have a protective effect on sepsis-induced ALI/ARDS. SAL is the main effective component of Rhodiola. It is speculated that it may further delay or reduce the occurrence of lung injury by inhibiting the release of inflammatory factors by reducing the inflammatory response of LPS-induced endotoxemia mice and improving the survival rate of mice^12,24,25^. In this study, it is found that LPS can induce the expression of miR-146a in the exosomes secreted by RLE-6TN cells to decrease, while SAL can reverse this reaction and significantly increase the content of miR-146a in the exosomes. In addition, in vitro experiments, it is found that the expression of miR-146, NF-kB, IRAK, TRAF6 and related inflammatory factors in NR8383 cells induced by LPS was higher than that in the control group, while SAL significantly reduced the expression of TLR4 mediated NF-kB inflammatory pathway and related inflammatory factors. In vivo test, the result was also confirmed that SAL can significantly improve the LPS-induced lung morphological abnormalities, and reduce the ALI score. The expression trends of NF-kB, IRAK, TRAF6 and related inflammatory factors in rat lung were also consistent with the results in vitro experiments. This indicates that LPS stimulation can lead to the decrease of miR-146a expression in exocrine body secreted by alveolar epithelial cells. Exocrine bodies absorbed by AM change the signal pathway of AM and activate them to produce inflammatory reaction. Although it is prove that SAL can further inhibit the expression of TLR4 mediated NF-kB inflammatory pathway and related inflammatory molecules in AM by strengthening the expression of miR-146a in exosomes secreted by pulmonary epithelial cells, in fighting against LPS-induced ALI/ARDS. However, the effect of different doses of SAL on ALI/ARDS needs further studies.

## Conclusion

In conclusion, SAL can inhibit the expression of NF-kB, IRAK, TRAF6 and related inflammatory molecules in AM by promoting the expression of miR-146a in exosomes secreted by pulmonary epithelial cells. At the same time, the evaluation indexes of ALI in rats saw significant improvement, which suggests that SAL has a protective effect on ALI/ARDS caused by sepsis, and likely to be used as a potential drug for the treatment of the disease. This study provides a new theoretical basis for the clinical treatment of ALI with SAL.

## Supplementary information

Supplementary Information.
