# Supplementary material for: Salidroside regulates inflammatory pathway of alveolar macrophages by influencing the secretion of miRNA-146a exosomes by lung epithelial cells
Source: Sci Rep. 2020 Nov 27;10:20750. doi: 10.1038/s41598-020-77448-6 (PMC7695860; doi:10.1038/s41598-020-77448-6)

**Title page**

**Title:** Salidroside Regulates Inflammatory Pathway of Alveolar Macrophages by Influencing the Secretion of miRNA-146a Exosomes by Lung Epithelial Cells

**Short title:** Salidroside Regulats Inflammatory Pathway of Alveolar Macrophages

Lanzhi Zheng^1^, Jianming Su^1^, Zhuoyi Zhang^1^, Lu Jiang^1^, Jinling Wei^1^, Xiaoyang Xu^1^, Shumin Lv^2*^

1 Emergency department, The First Affiliated Hospital of Zhejiang Chinese Medical University, Hangzhou City Zhejiang Province, 310006, China

2 Department of Cardiology, The First Affiliated Hospital of Zhejiang Chinese Medical University, Hangzhou City Zhejiang Province, 310006, China

**Corresponding author:**

Shumin Lv

Department of Cardiology, The First Affiliated Hospital of Zhejiang Chinese Medical University, Hangzhou City Zhejiang Province, 310006, China

Email: ShuminLv666@126.com

**Table S1: Primer sequences.**

| Genes | Primers（3’-5’） |
| --- | --- |
| TLR4 | F:ATCTCAGCAAAATCCCTCAT |
|  | R:AATCCAGCCACTGAAGTTGT |
| TRAF6 | F:AAGTTGCCGAGATGGAAGC |
|  | R:CCAGGGCTATGAATGACCAC |
| miR-146a | F:TGAGAACTGAATTCCATGGGTT |
|  | R:GGCCAACCGCGAGAAGATGTTTTTTTTT |
| NF-kB | F:TCTGTTTCCCCTCATCTTTCC |
|  | R:GCGTCTTAGTGGTATCTGTGCTT |
| IRKA1 | F:GCTCCCAGACCCATTCTGAG |
|  | R:CTCTGGGCTGGCTTGATGG |
| GAPDH | F:CCTCAAGATTGTCAGCAAT |
|  | R:CCATCCACAGTCTTCTGAGT |

**Figure 1B: As full as possible length gels and blots for Figure 1.**


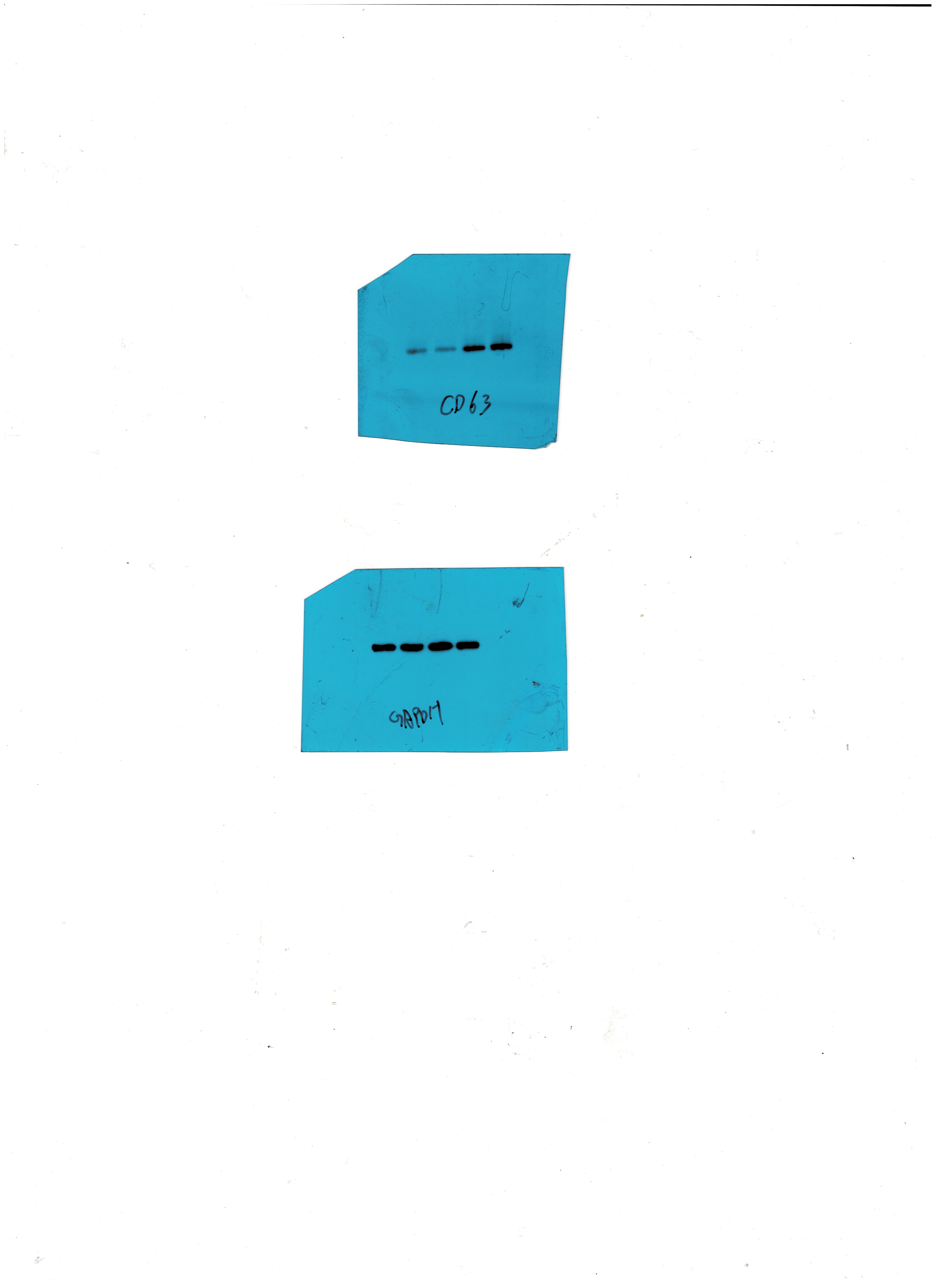


**Figure 2B: As full as possible length gels and blots for Figure 2.**


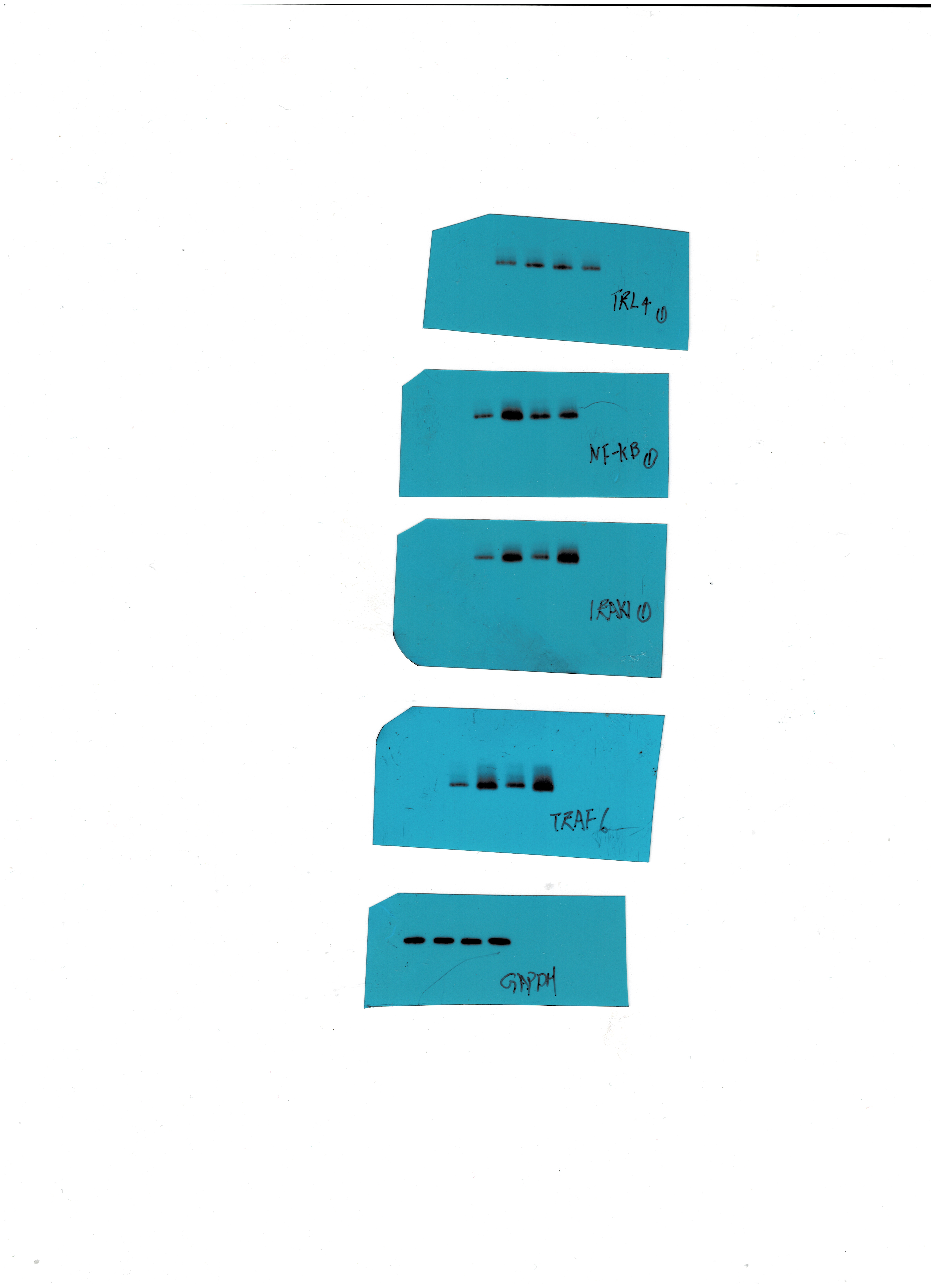


**Figure 4B: As full as possible length gels and blots for Figure 4.**


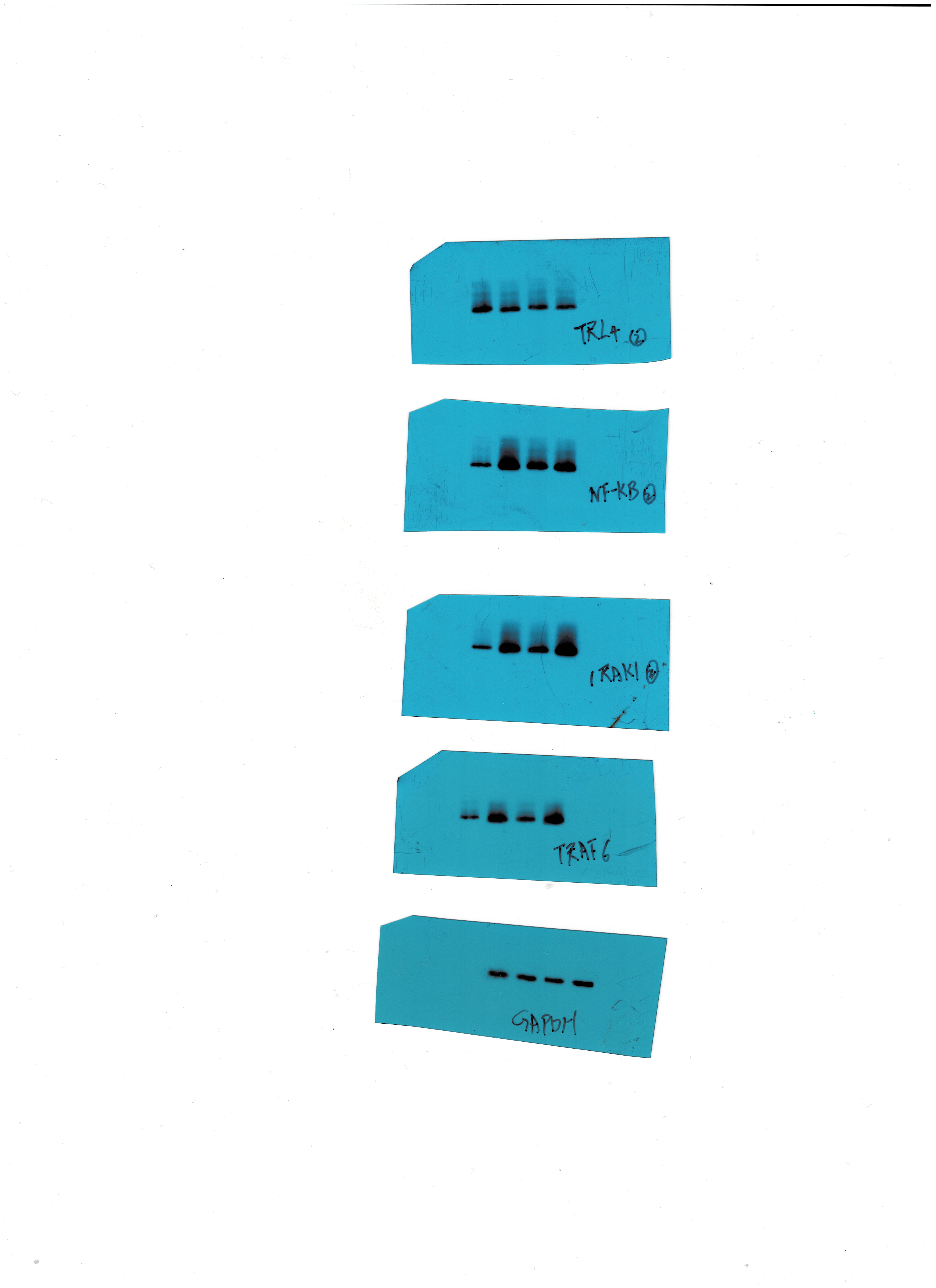

Supplement: Supplementary file 1 — Supplementary Information. [file 41598_2020_77448_MOESM1_ESM.doc]
